# Rapid Assessment of Price Instability and Paucity of Medicines and Protection for COVID-19 Across Asia: Findings and Public Health Implications for the Future

**DOI:** 10.3389/fpubh.2020.585832

**Published:** 2020-12-14

**Authors:** Brian Godman, Mainul Haque, Salequl Islam, Samiul Iqbal, Umme Laila Urmi, Zubair Mahmood Kamal, Shahriar Ahmed Shuvo, Aminur Rahman, Mustafa Kamal, Monami Haque, Iffat Jahan, Md. Zakirul Islam, Mohammad Monir Hossain, Munzur-E-Murshid, Santosh Kumar, Jaykaran Charan, Rohan Bhatt, Siddhartha Dutta, Jha Pallavi Abhayanand, Yesh Sharma, Zikria Saleem, Thuy Nguyen Thi Phuong, Hye-Young Kwon, Amanj Kurdi, Janney Wale, Israel Sefah

**Affiliations:** ^1^Strathclyde Institute of Pharmacy and Biomedical Sciences, University of Strathclyde, Glasgow, United Kingdom; ^2^Division of Clinical Pharmacology, Karolinska Institute, Karolinska University Hospital Huddinge, Stockholm, Sweden; ^3^School of Pharmacy, Sefako Makgatho Health Sciences University, Pretoria, South Africa; ^4^School of Pharmaceutical Sciences, Universiti Sains Malaysia, Penang, Malaysia; ^5^Unit of Pharmacology, Faculty of Medicine and Defence Health, Universiti Pertahanan Nasional Malaysia (National Defence University of Malaysia), Kuala Lumpur, Malaysia; ^6^Department of Microbiology, Jahangirnagar University, Dhaka, Bangladesh; ^7^Department of Orthodontics, Faculty of Dentistry, Bangabandhu Sheikh Mujib Medical University, Dhaka, Bangladesh; ^8^Integrated Sleep Disorders Center, McGuire VAMC/VCU Health, Richmond, VA, United States; ^9^RMC Hospital & Diagnostic Complex Ltd., Dhaka, Bangladesh; ^10^Finance & Account Division, Grameen Euglena, Dhaka, Bangladesh; ^11^Al-Manar Hospital Ltd., Modern Hospital Cumilla Ltd., Dhaka, Bangladesh; ^12^Human Resource Department, Square Toiletries Limited, Rupayan Center, Dhaka, Bangladesh; ^13^Department of Physiology, Eastern Medical College, Comilla, Bangladesh; ^14^Department of Pharmacology, Eastern Medical College, Comilla, Bangladesh; ^15^Department of Anatomy, Eastern Medical College, Comilla, Bangladesh; ^16^WISH2ACTION Project, Handicap International, Kurigram, Bangladesh; ^17^Department of Periodontology and Implantology, Karnavati University, Gandhinagar, India; ^18^Department of Pharmacology, All India Institute of Medical Sciences, Jodhpur, India; ^19^Department of Pediatric Dentistry, Karnavati University, Gandhinagar, India; ^20^Department of Conservative Dentistry and Endodontics, Rajasthan University of Health Sciences, Jaipur, India; ^21^Department of Pharmacy Practice, Faculty of Pharmacy, The University of Lahore, Lahore, Pakistan; ^22^Pharmaceutical Administration & PharmacoEconomics, Hanoi University of Pharmacy, Hanoi, Vietnam; ^23^College of Pharmacy, Seoul National University, Seoul, South Korea; ^24^Department of Pharmacology, College of Pharmacy, Hawler Medical University, Erbil, Iraq; ^25^Independent Consumer Advocate, Brunswick, VIC, Australia; ^26^Ghana Health Service, Keta Municipal Hospital, Pharmacy Department, Keta, Ghana; ^27^University of Health and Allied Sciences, School of Pharmacy, Pharmacy Practice Department, Volta Region, Ghana

**Keywords:** Bangladesh, community pharmacists, COVID-19, India, Malaysia, Pakistan, price rises, Vietnam

## Abstract

**Background:** Countries have introduced a variety of measures to prevent and treat COVID-19 with medicines and personal protective equipment (PPE), with some countries adopting preventative strategies earlier than others. However, there has been considerable controversy surrounding some treatments. This includes hydroxychloroquine where the initial hype and misinformation lead to shortages, price rises and suicides. Price rises and shortages have also been seen for PPE. Such activities can have catastrophic effects on patients where there are high co-payment levels and issues of affordability. Consequently, there is a need to investigate this further.

**Objective:** Assess changes in the availability, utilization and prices of relevant medicines and PPE during the pandemic among a range of Asian countries.

**Our approach:** Narrative literature review combined with interviews among community pharmacists to assess changes in consumption, prices and shortages of medicines and PPE from the beginning of March 2020 until end of May 2020. In addition, suggestions on ways to reduce misinformation.

**Results:** 308 pharmacists took part from five Asian countries. There was an appreciable increase in the utilization of antimicrobials in Pakistan (in over 88% of pharmacies), with lower increases or no change in Bangladesh, India, Malaysia and Vietnam. Encouragingly, there was increased use of vitamins/immune boosters and PPE across the countries, as well as limited price rises for antimicrobials in India, Malaysia and Vietnam, although greater price rises seen for analgesics and vitamin C/immune boosters. Appreciable price increases were also seen for PPE across some countries.

**Conclusion:** Encouraging to see increases in utilization of vitamins/immune boosters and PPE. However, increases in the utilization and prices of antimicrobials is a concern that needs addressing alongside misinformation and any unintended consequences from the pandemic. Community pharmacists can play a key role in providing evidence-based advice, helping to moderate prices, as well as helping address some of the unintended consequences of the pandemic.

## Introduction

### General and Asia

The coronavirus disease 2019 (COVID-19) pandemic was first identified in Wuhan, China, during December 2019, and by 27 September 2020 there were 32,731 million cases and over 990,000 deaths worldwide giving a case fatality ratio (CFR) among confirmed cases of 3.03% (1–3). This included over 6,721 million confirmed cases in the World Health Organization (WHO) South East Asian Region, including Bangladesh and India, with over 110,000 deaths, giving a CFR of 1.65% and the Western Pacific Region with over 600,000 reported cases and over 13,000 deaths giving a CFR of 2.187% by 27 September 2020 (1). The lower prevalence and mortality rates among the WHO Western Pacific countries, including Malaysia, Korea, and Vietnam, compared with Bangladesh, India, and Pakistan (Table 1), appears to reflect early proactive testing and lockdown policies, combined with other factors, building on the lessons learnt from earlier pandemics (Table 2), even with appreciable under-reporting in Bangladesh, India, and Pakistan (2, 4, 7, 8, 12, 14, 15, 37, 50, 52, 56, 57). A similar situation has been seen in Taiwan with the National Health Command Centre including the Central Epidemic Command Centre rapidly instigating educational activities, active tracking systems, accelerated production of personal protective equipment (PPE), lockdown, and quarantining measures (58, 59). By early April there were <400 confirmed cases in Taiwan among its 23 million citizens (58, 59), with only 513 confirmed cases by 29 September and seven deaths (60). We have also seen low prevalence and mortality rates among a number of African countries that instigated pro-active measures early compared with high income countries such as Western European countries and the USA (1, 61). Early engagement with citizens is crucial with preventing the spread of COVID-19 with countries such as Korea, Malaysia, Taiwan and Vietnam learning from earlier pandemics (58, 62). This compares with for instance Iran where the first cases of COVID-19 were confirmed on 19 February 2020 and by March 8 there were 6,566 confirmed cases and 194 deaths (63, 64). Some preventative measures were introduced Mid March to help reduce the spread of the virus, which included awareness campaigns, closing of educational facilities and disinfecting busy places (63–66) alongside a national screening programme but without widespread testing (64). However, stricter travel bans were only introduced at the end of March following travels during the Iranian New Year (64). This lack of early pro-activity may well have contributed to Iran having the highest prevalence rates (443,086) and deaths (25,394) due to COVID-19 in the WHO Eastern Mediterranean region by 27 September 2020 including Pakistan (Table 1) despite concerns with economic sanctions (1, 67, 68).

**Table 1:** Confirmed prevalence and mortality rates for COVID-19 in selected South East Asian and Western Pacific countries (27 September 2020).

**Country**	**Population size**	**First recorded case**	**Prevalence rates**	**Current deaths**	**CFR rate**
Bangladesh	164,689,383	Early March 2020 (4)	357,873	5,129	1.43%
India	1,380,004,385	30 January (5, 6)	5,992,532	94,503	1.58%
Malaysia	32,365,999	25 January (7, 8)	10,769	133	1.24%
Pakistan	220,892,340	25/26 February 2020 (9–11)	310,275	6,457	2.08%
South Korea	51,269,185	19 January 2020 (12, 13)	23,611	401	1.70%
Vietnam	97,338,579	First case 23 January; second wave 6 March (14–16)	1,069	35	3.27%

*NB: Population figures taken from Worldometer data for 2020 (17–19) and epidemiology data from WHO (1). CFR, case fatality rate*.

**Table 2:** Early activities instigated among selected Asian countries to help with prevention and treatment of COVID-19.

**Country**	**Ongoing activities including dates where known**
Bangladesh (summary) (4)	• First week of March 2020 – The Government started to postpone/cancel all mass gatherings • 15 March 2020 – The Government banned all flights coming from Europe except the United Kingdom and on 16 March imposed a 14-day obligatory quarantine on all travelers entering Bangladesh • 16/19 March 2020 - The Government closed all educational institutes and instructed local administrations to ban political and religious rallies as well as social and cultural gatherings • 23 March 2020 - The Government announced the closure of all public and private offices and on 25 March declared the enforcement of lockdown measures for 10 days effective from March 26, which was further extended • 12 June 2020 – The Government introduced the concept of risk zones for the prevention of COVID-19 • In addition, ongoing concerns with sufficient PPE; although gifts have helped
India	• 30 January 2020 – Surveillance strengthened at points of entry and in the community (6) • 6 February 2020 – The Government issued travel restrictions to China and anyone with a travel history from China from 15th January 2020 will be quarantined (20). The Ministry of Health instigated a 24 h/7 days-a-week disease alert helpline to provide information including clinical guidance (21, 22), with the textile industry producing PPE to address shortages (23) • 8 March 2020 – 52 laboratories were identified by ICMR for undertaking testing for COVID-19 (24), now increasing and all international passengers mandated to undergo universal medical screening (24) • 15 March 2020 - all movement suspended for foreigners through all Immigration Land Check Posts with only a few exceptions (Nepalese and Bhutanese nationals) • 22 March 2020: “Janata Curfew” introduced with 14 h lockdowns (21), all children under 10 and all elderly over 65 told to remain at home unless essential (25); Ministry of Pharma and Consumer Affairs instructed to take necessary action to regulate prices for PPE and other health related materials and to facilitate their availability in hospitals and the population (25) • 25 March 2020 - Further lockdown measures initiated for 21 days starting on 25 March (5, 21, 26), and extended to 3 May (21) • 14 April 2020 – Lockdown extended until 3 May 2020 (27), and further lockdown extended for 2 weeks from 4 May 2020; however, variable activities across districts depending on current infection rates (28)
Malaysia	• 6 February 2020 – Updated travel restrictions from travelers coming from infected Provinces in China (29) • February 2020 – Ministry of Health in Malaysia appreciably upgraded health facilities and diagnostic capacity including a 86% increment in diagnostics laboratory capacity, a 89% increment in ICU capacity and a 49% increase in the number of ventilators (from 526 to 1,034 units); in addition hospitalizing all COVID-19 positive patients (8) • 5 March 2020 – Expanded travel restriction list especially those coming from infected areas (7) • 16/18 March 2020 – Borders closed and Malaysian citizens not allowed to leave the country; all schools, universities and non-essential businesses also closed; and control of movement (7, 30, 31) • 25 March 2020 – Current movement restrictions extended until 14 April, with the army deployed from 22 March onwards to help enforce movement restrictions (7, 32) • 15 April 2020 – The Government announces it will jail movement violators (31) • 29 April 2020 – Lockdown measures eased, e.g., easing of social distancing rules (33) • 30 April 2020 – testing capacity increased up to 14,000 tests per day and soon up to 20,000 tests/day, building on the experience with other infectious diseases including dengue and tuberculosis (8) • 10 May – 4 week extension to existing restrictions on movement and business until 9 June (34) • Early June (9 June onwards) – Most restrictions lifted on businesses (35) Overall, increasing compliance with movement restriction orders is seen as a major reason why a decrease in new COVID-19 cases was seen in Malaysia from mid-April onwards (7)
Pakistan	• 13 March 2020 – National co-ordination committee formed and all colleges and universities closed – further extended till end May 2020 (9, 11, 36) • Mid-March 2020 – Quarantining of retuning travelers including those from religious festivals. However, system in some States over whelmed and becoming centers for infection with lack of facilities including hand sanitization (37, 38). In addition, start of sealing of borders (Iran and Afghanistan) (36) • Mid-March/17 March 2020 – Initially no national lockdown due to concerns with adults feeding themselves and their families if no longer working; however advice from the Ministry of Health on ways to reduce the spread of the virus including avoid public gatherings, regularly washing hands and social distancing including distancing from infected patients (38, 39) • Mid-March/April – Government offering financial support to citizens and businesses to help address financial concerns during the pandemic (9, 40) • 21 March 2020 – Pakistan banned International flights (41) • 24 March 2020 – National lockdown imposed urging citizens to stay indoors apart from food and medicines given rising prevalence rates and suspending railway operations until at least 31 March 2020. Lockdown aided by law enforcement officers and the military. Further extensions to 14 April 2020 (39, 41–43) • 9 May 2020 – Lockdown lifted principally in view of economic needs; however, concerns that lifted too soon (44, 45). More recently, targeting of “hot spots” to help control the spread (46, 47)
South Korea	• End January 2020 – Government establishing an emergency response committee (12) • 27 January 2020 – Korean Centre for Disease Control (CDC) directed private companies to help produce diagnostic reagents (12). By March 100,000 kits were shipped daily and by 24 April 2020 118 institutions were available to run diagnostic tests (12, 48). Screening centres were established outside of hospitals and other institutions to help track and trace the virus enabling the country to perform 300,000 tests per day by late March (12, 49) • 12 February 2020 – Quarantining required for travelers from Hong Kong and Macau (12) • 21 February – Designating hospitals in Daegu for treating COVID-19 patients, and building on this (49)
	• Mid-February to Early March – Initiatives to double the production of masks including companies re-purposing their garment factories, with the Government subsequently purchasing 80% of mask supplies from Korean manufacturers, banning exports and setting price limits thereby helping to avert PPE shortages (12, 49, 50) • 7 March 2020 – GPS-App to go live to help enforce quarantining (12) • 23 March 2020 – Enhanced social distancing campaign (12) • 1 April 2020 – Required 14-day quarantine for all travelers with self-reporting App • 8 April 2020 – Seoul closes bars, etc. (12) • 3 May 2020 – Relaxation of social distancing (50) • 11 May 2020 – School opening delayed apart from high school seniors (12) South Korea expanded its Epidemic Intelligence Service to help with early detection and to keep rates of infection low (12)
Vietnam	Figure 1 contains details of time lines and cases. In summary: • 10 January 2020 – Government reinforced temperature and health status screening at border gates for passengers arriving from Wuhan (51) • 20 January 2020 - 22 hospitals chosen for the treatment of suspected COVID-19 (51) • 31 January 2020 – All schools to remain closed (14, 51) • End January – Early February 2020, Government Taskforce Group under the Vice Prime-Minister on COVID-19 formed to direct and coordinate activities among the ministries (16, 52) with early measures including suspending flights from China and other epidemic areas, limiting crowds especially at festivals, suspending festivals not yet opened, asking people to wear masks in public places and limit travel (14, 16, 51). The media in Vietnam was heavily involved in conveying Government messages regarding prevention and other activities including SMS texts (16, 51) with concerns that some people are ignoring warnings • 7 February 2020 – Testing enhanced by the development of a test kit at Hanoi University of Science and Technology with further kits developed by Vietnam Academy of Science and Technology as well as other Universities from 3 March onwards (14) with 120 testing sites up and running by May 2020 • February 2020 – First clinical guideline on diagnosis and treatment of COVID-19 patients introduced and updated in March as more data became available (16) • 28 February 2020 - Mandatory 14-day quarantine for all travelers entering Vietnam from a COVID-19 affected country (51) • 19 March 2020 – Mandatory use of the Hanoi Smart City app to monitor the health and movement of recovered confirmed cases, suspected cases, and people under quarantine (51) • 20 March 2020 – Strict quarantining for anyone entering the country (16) • 1 April 2020 – National lockdown measures introduced for 15 days and subsequently extended to 21 days in 28 out of 63 provinces (14, 16) • 13 April 2020 – Pharmacists are requested to ask patients buying medicines for acute respiratory infections such as coughs, fever, shortness of breath and colds to make a health declaration (53) • 23 April 2020 – Start of lifting of lockdown measures (54) In addition, pushing for self-sufficiency in the production of medicines and other technologies to help with future pandemics (55)

**Figure 1:**
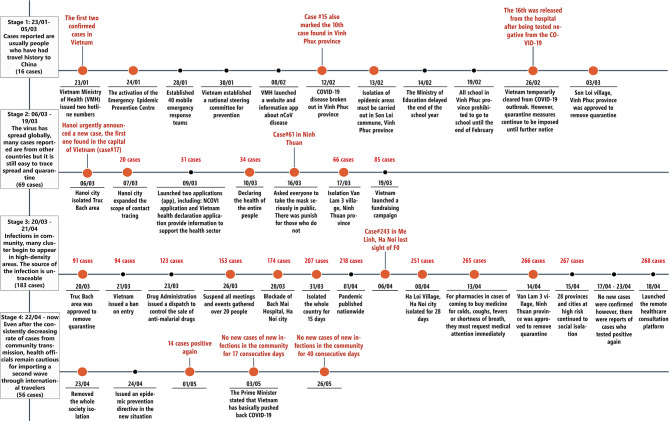
Time lines of prevalence rates and activities in Vietnam to reduce the spread of COVID-19 and its consequences.

The active tracking systems in for instance in Taiwan and Vietnam compare with Pakistan where by 3 April 2020 only 30,308 laboratory tests had been conducted of which 2,450 tested positive (9). We are aware though that over-crowding as well as a lack of clean water and sanitation in Bangladesh, India, and Pakistan will increase infection rates unless adequately addressed compared with higher income countries (4, 5, 69–71). This is in addition to ongoing challenges with the subclinical manifestation of COVID-19 across all populations (72).

Adequate prevention and management of patients with COVID-19 is also hindered in Bangladesh, India and Pakistan compared with Malaysia, South Korea, and Vietnam, with high levels of patient co-payments to purchase medicines and other treatments, which can be catastrophic for families if members become ill (4, 73, 74). For instance in Bangladesh, an appreciable number of households are forced to sell their assets or borrow money to fund care when family members become ill affecting care delivery (4). This typically includes the cost of medicines as seen in patients with type 2 diabetes (T2DM), where medicine costs account for 83.5% of total direct medical costs, which is typically out-of-pocket (4, 75). In India, up to 39 million people have been pushed into poverty each year due to healthcare co-payments; however, this is beginning to change with ongoing reforms to provide health insurance coverage for up to 100 million families in poverty (73, 76–78). In addition, there have been recent initiatives in India to control the prices of essential medicines as well as encourage the use of generic medicines where possible (79, 80). Alongside this convergence of healthcare spending toward OECD average spending levels with movement toward universal healthcare in the coming years although affordability of care for the poor will remain a continuing challenge (81–84).

In Pakistan, there have been concerns with healthcare management at the provincial level following devolution, although this is not universal as seen with the recent COVID-19 pandemic (40, 74, 85). This is a concern with over 40% of the population currently living below the poverty line, and high rates of co-payments at over 78% of total healthcare expenditure with potentially catastrophic consequences if family members become ill (74, 86–88). The situation has been made worse in Pakistan in recent years with a rise in both infectious and non-infectious diseases. In 2019, there was a rise in the prevalence of dengue, HIV/AIDS, malaria, measles and typhoid compared to previous years, enhanced by consumption of contaminated water and food as well as environmental pollution even before the COVID-19 pandemic (89). Consequently, there are concerns that COVID-19 could further overwhelm the healthcare system. Having said this, knowledge, attitude and practices toward COVID-19 appeared satisfactory among healthcare professionals in Pakistan with a high percentage having a positive attitude toward prevention and management; however, there were some misconceptions that need to be addressed going forward (90, 91). There were concerns though about the extent of preventative practices among university students and employees despite good knowledge and attitudes toward COVID-19 (92).

There has also been a lack of intensive care unit (ICU) beds and ventilators among public hospitals in Bangladesh to treat patients with severe COVID-19, with a similar situation in India and Pakistan (4, 5, 9, 42, 89, 93), affecting care delivery. We have seen countries in Africa respond to the challenge of lack of ventilators through instigating local design with the help of universities, along with increased local production of hand sanitisers and other preventative measures including face shields and splash masks to reduce the spread of the virus as well as improve management once patients are in hospital (61, 94) providing direction to others. Local production of essential preventative equipment is likely to grow across countries to reduce future shortages (61, 62). Compounding the situation in Bangladesh, India, and Pakistan, certainly with respect to available resources to fund prevention and treatment of COVID-19, are also high prevalence rates of non-communicable diseases (NCDs) including coronary vascular disease (CVD), hypertension and diabetes (4, 74, 87, 95–97). This will necessitate changing models of care with increasing use of all healthcare professionals, exacerbated by the current situation with COVID-19 (98–101). This is in addition to continuing concerns with funding treatments for other infectious diseases, which, as mentioned, appear to be increasing in Pakistan (4, 76, 89, 102, 103). Alongside this, there appears a general shortage of medicines to treat diseases in Pakistan not help by concerns with affordability among manufacturers (104, 105).

The situation is different in Malaysia with a public healthcare system funded via general taxation in addition to a private healthcare system, which includes dispensing general practitioners (106, 107). The cost of medicines still accounts though for an appreciable proportion of total costs in the public healthcare system in Malaysia. There is though an active procurement process as well as demand-side measures encouraging the prescribing of generics as part of ongoing measures to help contain costs (106) similar to ongoing measures in India (79). The final agreed price for essential medicines in the public system should also ideally be no more than three times the International Reference Price (IRP) (106). However, there are currently a lack of price controls for medicines and other technologies in the private sector including mark-ups, which can add to the cost of medicines (108, 109). This lack of control is seen in the price of medicines for treating ischaemic heart disease (IHD) where the lowest price of generics or originator brands to treat IHD were 10.77 and 24.09 times, respectively, above IRPs, and can make these medicines unaffordable for the average worker who purchases them in community pharmacies rather than attending primary healthcare clinics (PHCs) with their long waiting times (110–112). Currently in Malaysia, community pharmacists have a lesser of a role in dispensing medicines than seen in many other countries due to an appreciable number of dispensing physicians (113, 114). This needs to be addressed especially in patients with chronic diseases such as type 2 diabetes (T2DM) to improve their care given appreciably increased rates in recent years (115–117). In the meantime, pharmacists do have an important role in the management of acute respiratory tract infections such as coughs and colds, for gastro-intestinal ailments (118), as well as minor ailments generally (119) in Malaysia, helped by the long waiting times to see healthcare professionals in PHCs (111).

Social insurance coverage has increased in Vietnam in recent years to reduce the burden of out-of-pocket payments (120, 121). However, out-of-pocket payments still exist, with health insurance only covering part of the costs with potentially distressing consequences for a minority of families when members become ill (120, 122). As a result, community pharmacists continue to play a key role in managing diseases in Vietnam with for instance over 80% of people typically visiting a pharmacist first for their illness. These scenarios are common especially in rural areas to conserve costs for patients and enhance profits for pharmacists; however, rates of self-purchasing of medicines are lower than before enhanced by increased social insurance (113, 123–125). Despite this, the costs of medicines can still be a concern in Vietnam with high costs vs. IRP (126).

Overall across Asia, pharmacies are often the main source of healthcare for patients given lack of waiting times, extended opening hours, and high co-payment levels (103, 115, 127–131), mirroring the situation in other countries (132–134). Consequently, community pharmacists have a key role to play in current and future pandemics.

### Management Strategies to Prevent and Treat COVID-19 – General and Across Asian Countries

COVID-19 is transmitted from person to person principally through respiratory droplets and aerosol transmission forming the basis of preventative measures across countries (Table 2) (4, 135–141). Preventative messages appear to be accepted by the population with for instance 85.8% of citizens in Pakistan in a recent survey stating they regularly wear face masks and 88.1% undertake handwashing to try and prevent the spread (10). However, as mentioned, there were concerns about the extent of preventative practices among university students and employees (92).

Current evidence suggests increased morbidity and mortality from COVID-19 is associated with underlying health conditions including hypertension, CVD, diabetes, smoking, shortness of breath, chronic obstructive pulmonary disease (COPD) and blood types (142–149). Ethnicity may also be important with patients in the United Kingdom of South Asian origin at appreciably increased risk of dying from COVID-19 vs. those of white ethnicity (150–154), which is a concern in Asia along with high rates of smoking and growing rates of chronic NCDs including CVD and diabetes (4, 95, 97, 155–157). However, currently there appears to be no cure for patients with COVID-19. A number of medicines have been proposed, rejected or are still undergoing trials (4, 158–161). This means that preventative measures are very important to reduce morbidity and mortality from COVID-19. In addition, predictive models in hospitals to better target resources and treatments (162, 163).

The most promising treatment to date appears to be dexamethasone, shown in the UK Recovery Trials to reduce the number of deaths in ventilated patients and in those receiving oxygen only (164). Remdesivir has also shown encouraging results despite earlier concerns in an underpowered study (165–167), although there appears less benefit in patients with moderate COVID-19, and recent studies are questioning its role in treatment (168, 169). Triple antiviral therapy has also shown promise although patient numbers in the trial are small (170). Most controversy surrounds the use of chloroquine or hydroxychloroquine with or without azithromycin for prevention or treatment following initial studies in China (171–173). There were concerns about the lack of comparisons in the initial studies alongside potential harm including cardiac side-effects (172, 174, 175). A number of recent studies have endorsed these concerns including the UK Recovery Trial (171, 176–178), resulting in the WHO and the National Institute of Health (NIH) in the US halting the use of hydroxychloroquine in their studies (161, 167, 177, 179, 180). However, hydroxychloroquine is still recommended for prophylaxis in India with Chatterjee et al. (181) demonstrating that hydroxychloroquine was associated with a significant reduction in the chances of healthcare workers getting COVID-19 (21, 181, 182). The Government and others in Malaysia also initially endorsed hydroxychloroquine helped by additional supplies from India (183), with continued endorsement at lower doses in early June (8, 184, 185), with the Government in Pakistan banning exports for fear of shortages (186), although later reversed (187). There have also been ongoing studies with chloroquine in Vietnam (188). However, hydroxychloroquine is no longer recommended in Malaysia following recent analysis of data collected from 500 cases, which showed no effect (189).

There have been similar controversies surrounding lopinavir/ritonavir, which is endorsed in India but now dropped from the WHO Solidarity study and the UK Recovery study due to concerns with its effectiveness (21, 161, 190). Consequently, there is a need to ensure that recommended strategies are evidence based given current controversies and redactions (161, 174, 191, 192). This is because misinformation can have devastating consequences, which include increasing the cost of medicines as well as increasing hospitalisations and deaths from poisoning from hydroxychloroquine (4, 193–196). In addition, diverting scarce monies away from purchasing necessary personal protective equipment (PPE) and monies to treat priority infectious and non-infectious diseases. Table 2 documents current prevention and other strategies among selected Asian countries to help provide future direction, building on published activities for Bangladesh (4).

Unintended consequences of COVID-19 include concerns that patients with NCDs are not attending follow-up clinics and not receiving their medicines (4, 99), which is an increasing concern among Asian countries including India (98, 197). In addition, concerns that the mental health of the population regarding their emotional, psychological, and social impact, is being adversely affected by the pandemic. This is likely to be the case among Asian countries with cultures highly dependent on socialization in terms of support and connectedness, with such activities appreciably affected by lockdown and other measures (198). However, we are aware that issues of mental health are being addressed through increased telemedicine approaches in lower- and middle-income countries (LMICs) (199), and this is likely to continue. Restrictions on movement with lockdown measures, as well as concerns surrounding COVID-19 transmission, have also reduced immunization rates in India and Pakistan in recent months, which also needs addressing (200, 201). Community pharmacists can help here including enhancing adherence to medicines in patients with chronic NCDs as well as give guidance and public health education on prevention and possible treatments for patients with suspected COVID-19 (4, 128, 202–205).

Pharmacists and others can also help balance demand and supply of medicines, which is essential in countries where there are high patient co-payment levels and concerns with medicine availability (4, 114). This is particularly important if shortages lead to high prices for pertinent medicines and PPE with potentially catastrophic consequences for families.

### Study Objectives

Consequently, we believe there is a need to study the availability and prices of suggested medicines and PPE to prevent and treat COVID-19 among pharmacies and drug stores in Asian countries with high rates of both infectious and non-communicable diseases, as well as issues regarding co-payments to provide future direction. We have published on these issues in Bangladesh, and we have seen price rises in other LMICs (4, 206–208). We now wish to compare and contrast the findings in Bangladesh with India and Pakistan with similar high co-payment levels and high rates of both infectious and non-infectious diseases, although with price controls in India (79), along with Malaysia and Vietnam where community pharmacists are active especially for minor ailments. In addition, where hydroxychloroquine has also been endorsed for the management of patients with COVID-19 certainly initially.

In addition, contrast countries where pro-active strategies initially (Table 2) appear to have appreciably lowered prevalence and mortality rates from COVID-19 (Korea, Malaysia and Vietnam) vs. Bangladesh, India, and Pakistan despite low levels of testing in these countries certainly initially (Table 1). The combined findings can help provide future direction to all key stakeholder groups.

## Materials and Methods

We adopted a similar multiple strategy approach to the paper of Haque et al. discussing activities in Bangladesh (4). This included updated information from a pragmatic review of the literature combined with a questionnaire survey among community pharmacies and drug store owners in India, Malaysia, Pakistan, and Vietnam, building on the findings in Bangladesh, to assess the current situation regarding usage patterns, prices, and availability of carefully selected medicines that could potentially be used in the management of COVID-19, as well as PPE in most countries, soon after the start of the pandemic (4). We also included herbal medicines in Pakistan and Vietnam as we are aware of their use in patients with COVID-19 among a number of LMICs despite concerns (209, 210). Medicines included pertinent antimalarials such as hydroxychloroquine, antibiotics such as azithromycin, analgesics (general including paracetamol), vitamins and immune boosters such as vitamin C as well as PPE including face masks (Box 1). We just asked for impressions in the first instance for changes in utilization, prices, and shortages, from early March to end of May 2020 if this was the only information available due to issues of confidentiality (4). The baseline was early 2020, i.e., just before active preventative measures in a number of the countries (Table 1). More specific data on actual changes in utilization and prices was asked if this was available; however, this did not include asking the pharmacists to break down any changes in utilization patterns and prices per month as this was deemed too problematic for this initial study.

Box 1 **Open ended questions to community pharmacists in India regarding pertinent medicines and equipment to prevent and treat COVID-19.**

- Country?
- What changes in purchasing/utilization patterns have you noticed in your pharmacy from the beginning of March until the end of May 2020 for antimalarials (hydroxychloroquine), antibiotics (e.g., azithromycin and co-amoxiclav), multivitamins including Vitamin C and analgesics? The baseline is early 2020. Please base this information on invoices where possible or other information sources; otherwise, impressions
- What changes in the prices have you noticed for pertinent medicines from the beginning of March until end May 2020 for antimalarials, antibiotics, multivitamins and analgesics (based on invoices or other information sources where possible). The baseline is early 2020. Please base this information on invoices where possible or other information sources; otherwise, impressions
- Have there been any shortages for antimalarials, antibiotics, multivitamins and analgesics from the beginning of March until end May 2020 in your pharmacies? If so, what has been the extent if known?
- Similarly, for PPE including face masks, hand sanitisers and thermometers (utilization, prices, and shortages) from the beginning of March until the end May 2020 (baseline early 2020) – based again on invoices or other information sources/impressions
- Similarly for herbal medicines in Pakistan and Vietnam
- What suggestions can you give the authorities to address misinformation regarding the current pandemic (if pertinent) as well as any inappropriate self-medication with antimicrobials for future pandemics given current concerns?

Convenience sampling was used to select pharmacists through emails, telephone contact, personal contacts and other mechanisms. Similar to the initial study in Bangladesh (4), there was no sample size calculation as there was no previous data to base calculations upon at the start of the study. In addition, the studies undertaken in Malaysia, Pakistan, and Vietnam were pilot studies to help determine the need for additional studies. All questions were again open ended with data captured on Excel spreadsheets (Box 1). A more detailed description of the questionnaire can be found in Haque et al. (4). The replies from the community pharmacists were collated where possible into logical bands for comparisons between countries including the initial analysis from Bangladesh (4). These bands were not pre-defined as this was an exploratory study, with changes in prices based on local prices and not converted into a single currency such as US dollars using current exchange rates since ascertaining actual prices was not an objective of this study. The changes in utilization and prices were absolute changes during the time period of the study. Suggested strategies going forward for all key stakeholder groups also builds on the combined experiences of the co-authors. We have successfully used this approach before to provide future direction in LMICs (4, 125, 211–215).

We believed that there would be price rises and shortages in other countries apart from Bangladesh. However, the nature and extent would depend on ongoing programmes within the country (Table 2) including greater price controls in India and Pakistan (79, 105).

Ethical approval for this study was not required according to national legislation and institutional guidelines. However, as before, all pharmacists freely provided the requested information having been given the opportunity to refuse to participate if wished. This is in line with previous studies undertaken by the co-authors in this and related areas including analysis of policies to enhance the rationale use of medicines and biosimilars, pricing policies and issues surrounding generics, which involved direct contact with health authority personnel and other key stakeholders (4, 125, 212, 216–219).

## Results

Table 3 provides details of the number of pharmacists and drug store owners taking part including the pilot studies in Malaysia and Vietnam. There was a low refusal rate in Bangladesh (36.1%), India (18%), and Malaysia (0%), with pilot studies conducted among selected pharmacies in Pakistan and Vietnam.

**Table 3:** Details of pharmacists and drug stores owners across the countries.

**Country**	**Number of pharmacies/Drug stores**
Bangladesh	170
India	111
Malaysia	12
Pakistan	9
Vietnam	6
**Total**	308

We will first report on changes in utilization patterns across the studied countries before reporting on any price changes and shortages.

### Utilization

Table 4 depict changes in the utilization patterns for the various medicines, vitamins and PPE from the beginning of March until the end of May 2020 among the studied countries. Encouragingly, there was no change or decreased utilization of antimalarials in an appreciable number of pharmacies in Bangladesh (51.2%), Malaysia (83.3%) and Vietnam, with no change in 45% of pharmacies in India, contrasting with Pakistan (no change in only 11.1%). There were differences in the utilization patterns with antibiotics with increases seen in Bangladesh (70.6% of stores) and Pakistan (100% of pharmacies) compared with only 42.3% in India and little or no increase in either Malaysia or Vietnam (Table 4).

Table 4Changes in the utilization for medicines and PPE between beginning March 2020 and end May 2020 among pharmacies across the countries (baseline = early 2020; *n* = number unless specified).

**Table:** 

**Change**	**Bangladesh**					**India**				
	**AM**	**AB**	**AG**	**Vit C**	**PPE**	**AM**	**AB**	**AG**	**Vit C**	**PPE**
Decrease/No demand (*n*)	9	5	0	0	2	25	23	21	1	0
No change (*n*)	78	45	4	16	6	25	41	15	10	2
Increase (not specified) to slight increase (*n*)	73	104	142	140	139	59	35	63	81	54
High increase to under 1.5-fold increase	1	9	15	5	10	0	0	0	0	0
1.5- to 3-fold increase (*n*)	9	7	9	9	13	1	5	3	11	3
3- to 5-fold increase (*n*)	0	0	0	0	0	1	1	6	0	3
Above 5-fold increase (*n*)	0	0	0	0	0	0	6	3	8	49
Total number	170	170	170	170	170	111	111	111	111	111
% increase	48.8	70.6	97.6	90.6	95.3	55.0	42.3	67.6	90.1	98.2
% no change/decrease	51.2	29.4	2.4	9.4	4.7	45.0	57.7	32.4	9.9	1.8

**Table:** 

**Change**	**Malaysia**					**Pakistan**					**Vietnam**					
	**AM**	**AB**	**AG**	**Vit C**	**PPE**	**AM**	**AB**	**AG**	**Vit C**	**HM**	**AM**	**AB**	**AG**	**Vit C**	**HM**	**PPE**
Decrease/No demand (*n*)	0	0	0	0	0	0	0	0	1	0	0	6	4	2	6	0
No change (*n*)	10	10	7	5	0	1	0	5	0	6	6	0	0	0	0	0
Increase (not specified) to slight increase (*n*)	0	0	4	0	2	5	7	3	3	3	0	0	2	4	0	0
High increase to under 1.5-fold increase (*n*)	1	1	0	0	0	2	1	1	4	0	0	0	0	0	0	6
1.5- to 3-fold increase (*n*)	1	1	1	4	0	1	1	0	1	0	0	0	0	0	0	0
3- to 5-fold increase (*n*)	0	0	0	3	8	0	0	0	0	0	0	0	0	0	0	0
Above 5-fold increase (*n*)	0	0	0	0	2	0	0	0	0	0	0	0	0	0	0	0
Total number	12	12	12	12	12	9	9	9	9	9	6	6	6	6	6	6
% increase	16.7	16,7	41.7	58.3	100.0	88.9	100.0	44.4	88.9	33.2	0.0	0.0	33.3	66.7	0.0	100.0
% no change/decrease	83.3	83.3	58.3	41,7	0.0	11.1	0.0	55.6	11.1	66.7	100.0	100.0	66.7	33.3	100.0	0.0

*AM, antimalarial; AB, Antibiotic; AG, analgesics; Vit C, Vitamin C and other vitamins and immune boosters; HM, herbal medicines; PPE, Face masks, thermometers and hand sanitisers. No change also includes situations where medicines were not dispensed without a prescription or not dispensed in community pharmacies (antimalarials and antibiotics)*.

There were though appreciable increases in the utilization of analgesics and vitamin C/immune boosters in both Bangladesh and India, and to a lesser extent in Malaysia and Vietnam, with increased use of vitamin C/immune boosters also seen in Pakistan during the study period. Encouragingly, there were increases in the utilization of PPE across all countries where documented, some of which was substantial in line with recommendations. Encouragingly as well, no increased use of herbal medicines in Pakistan contrasting with Vietnam. It is likely that we will continue to see increases in the utilization of vitamins/immune boosters and PPE across countries if COVID-19 infection rates increase following easing of any lockdown measures.

### Price Changes

Table 5 depicts price changes for pertinent medicines and PPE during the study period. Encouragingly, there were limited price increases in Malaysia and Vietnam for antimalarials and antibiotics, with greater price rises seen for analgesics and vitamin C/immune boosters. There were also very limited increases in prices in India and Pakistan enhanced by price control measures (79, 80, 105). As expected, price rises were seen in over 90% of pharmacies for PPE across studied countries, with substantial price rises seen particularly in Bangladesh.

Table 5Changes in the prices of medicines and PPE between beginning March 2020 and end May 2020 among pharmacies across the countries (baseline = early 2020; *n* = number unless specified).

**Table:** 

**Change**	**Bangladesh**					**India**				
	**AM**	**AB**	**AG**	**Vit C**	**PPE**	**AM**	**AB**	**AG**	**Vit C**	**PPE**
Decrease/Not available (*n*)	4	0	0	0	1	0	0	1	0	0
No change (*n*)	81	111	93	88	7	93	102	84	51	10
Increase (not specified)/ slight increase (*n*)	56	44	58	73	110	12	5	21	51	96
High increase up to 2-fold increase (*n*)	20	14	18	9	34	0	0	0	0	0
2- to 4-fold increase (*n*)	9	1	1	0	15	1	4	5	6	2
Above 4-fold increase (*n*)	0	0	0	0	3	5	0	0	3	3
Total number	170	170	170	170	170	111	111	111	111	111
% increase	50.0	34.7	45.3	48.2	95.3	16.2	8.1	23.4	54.1	91.0
% no change/decrease	50.0	65.3	54.7	51.8	4.7	83.8	91.9	76.6	45.9	9.0

**Table:** 

**Change**	**Malaysia**					**Pakistan**					**Vietnam**					
	**AM**	**AB**	**AG**	**Vit C**	**PPE**	**AM**	**AB**	**AG**	**Vit C**	**HM**	**AM**	**AB**	**AG**	**Vit C**	**HM**	**PPE**
Decrease/Not available (*n*)	0	0	0	0	0	0	0	0	0	0	6	0	0	0	0	0
No change (*n*)	10	12	7	7	0	8	7	8	7	8	0	5	4	5	6	0
Increase (not specified)/slight increase (*n*)	0	0	4	0	4	1	2	1	2	1	0	1	2	1	0	6
High increase up to 2-fold increase (*n*)	2	0	0	3	0	0	0	0	0	0	0	0	0	0	0	0
2- to 4-fold increase (n)	0	0	1	0	2	0	0	0	0	0	0	0	0	0	0	0
Above 4-fold increase (n)	0	0	0	2	6	0	0	0	0	0	0	0	0	0	0	0
Total number	12	12	12	12	12	9	9	9	9	9	6	6	6	6	6	6
% increase	16.7	0.0	41.7	41.7	100.0	11.1	22.2	11.1	22.2	11.1	0.0	16.7	33.3	16.7	0	100.0
% no change/decrease	83.3	100.0	58.3	58.3	0.0	88.9	77.8	88.9	78.8	88.9	100.0	83.3	66.7	83.3	100.0	0.0

*AM, antimalarial; AB, Antibiotic; AG, analgesics; Vit C, Vitamin C and other vitamins and immune boosters; HM, herbal medicines; PPE, Face masks, thermometers, and hand sanitisers*.

### Medicine and Prevention Shortages

Perhaps not surprisingly, shortages of a number of medicines were seen (Table 6). This was especially the case for antimalarials in Bangladesh, India, Malaysia, and Pakistan, with shortages of antibiotics principally seen in Malaysia and Pakistan. Shortages of PPE were also seen among the countries studied reflecting ongoing preventative strategies among the countries (Table 2).

Table 6Shortages of medicines and PPE between beginning March 2020 and end May 2020 among pharmacies across the countries (baseline = early 2020; *n* = number unless specified).

**Table:** 

**Change**	**Bangladesh**					**India**				
	**AM**	**AB**	**AG**	**Vit C**	**PPE**	**AM**	**AB**	**AG**	**Vit C**	**PPE**
Available/No shortages (*n*)	78	140	129	105	34	33	100	109	84	13
Shortages (unspecified) (*n*)	63	18	36	57	114	25	9	0	2	43
Not available (*n*)	20	2	1	0	8	0	0	0	0	0
Shortages–some of the time (*n*)	9	10	4	8	14	53	2	2	25	55
Total number	170	170	170	170	170	111	111	111	111	111
% Available/no shortages	45.9	82.4	75.9	61.8	20.0	29.7	90.1	98.2	75.7	11.7
% Shortages (% of total)	54.1	17.6	24.1	38.2	80.0	70.3	9.9	1.8	24.3	88.3

**Table:** 

**Change**	**Malaysia**					**Pakistan**					**Vietnam**					
	**AM**	**AB**	**AG**	**Vit C**	**PPE**	**AM**	**AB**	**AG**	**Vit C**	**HM**	**AM**	**AB**	**AG**	**Vit C**	**HM**	**PPE**
Available/No shortages/not dispensed (*n*)	4	5	5	5	2	0	1	8	2	9	6	4	6	6	6	0
Shortages/stock outs (*n*)	8	7	7	7	10	5	6	1	4	0	0	2	0	0	0	0
Shortages – some of the time (*n*)	0	0	0	0	0	3	1	0	3	0	0	0	0	0	0	6
Decreased availability (*n*)	0	0	0	0	0	1	1	0	0	0	0	0	0	0	0	0
Total number	12	12	12	12	12	9	9	9	9	9	6	6	6	6	6	6
% Available/ no shortages	33.3	41.7	41.7	41.7	16.7	0.0	11.1	88.9	22.2	100.0	100.0	66.7	100.0	100.0	100.0	0.0
% Shortages (% of total)	66.7	58.3	58.3	58.3	83.3	100.0	88.9	11.1	77.8	0.0	0.0	33.3	0.0	0.0	0.0	100.0

*AM, antimalarial; AB, Antibiotic; AG, analgesics; Vit C, Vitamin C and other vitamins and immune boosters; HM, herbal medicines; PPE, Face masks, thermometers and hand sanitisers*.

### Potential Ways Forward to Address Misinformation and Enhance Appropriate Use of Medicines and Equipment Across Sectors

Possible strategies to address concerns regarding the management of COVID-19 and any unintended consequences especially in Bangladesh and India are included in Table 7. This builds on previous suggestions for Bangladesh only (4).

**Table 7:** Key activities among stakeholder groups to improve prevention and management of patients with COVID-19.

**Stakeholder group**	**Suggested activities**
Government	• Encourage early preventative measures to reduce the spread of any virus during future pandemics – building on the successes to date in Malaysia, South Korea, and Vietnam vs. Bangladesh, India and Pakistan (Tables 1, 2) • As part of this, utilize social media and other platforms to rapidly disseminate information regarding suggested activities to prevent the spread of viruses; however, mindful of the likely situation regarding the ability to social distance and the lack of clean water in a number of households in LMICs. This builds on the findings of Hayat et al. (10) among citizens in Pakistan where following communication from the Government, in social media and other avenues, 77.0% of those surveyed believed COVID-19 could be controlled successfully with practices of wearing masks (85.8%) and handwashing (88.1%) common among participants (10) • Encourage an evidence-based approach for decision making and recommendations given the controversies that still surround hydroxychloroquine (India) and Malaysia (until recently) as well as for lopinavir/ritonavir. In addition, the redaction of a number of recent papers relating to COVID-19 • Alongside this, instigate measures to reduce the level of misinformation and its effects on diverting scarce resources away from funding medicines in other priority infectious and non-infectious diseases – in line with advice and recommendations from the Council for International Organisations of Medical Sciences (220). As part of this, explore the opportunity for financial consequences for companies spreading false misinformation and claims regarding potential treatments – building on activities in other countries (221, 222) • Continue with ongoing and planned programmes to improve the management of patients with existing chronic NCDs as well as other infectious diseases to minimize unintended consequences arising from activities to reduce the spread of COVID-19. Alongside this, instigate activities to help reduce mental health issues, including stigma, arising from COVID-19 • Similarly, for vaccination programmes – seek to address current concerns with reduced rates as a result of the pandemic and lockdown measures • Instigate/enforce measures to reduce inappropriate self-purchasing of medicines including antimalarials and antibiotics where concerns – building on successful measures in other countries (125, 223) • Instigate measures to enhance the local production of medicines and PPE to address current shortages and keep prices increases to a minimum. Investigate the potential for formal price controls building on examples in e.g., Pakistan • When pertinent, adopt a phased approach to any easing of lockdown and other measures, with rapid re-introduction of lockdown measures if needed (224)
Physicians	• Instigate a policy of evidence-based medicine in all aspects of care delivery starting in medical school and continuing post qualification given the ongoing controversies surrounding the use of hydroxychloroquine, lopinavir-ritonavir and remdesivir in the prevention and management of COVID-19 • As part of this, continue to ensure recommended treatments are evidence based through postgraduate training and other continuous professional development activities post qualification • Continue to encourage where possible appropriate identification and management of patients with NCDs including CVD and diabetes, which includes encouraging adherence to prescribed medicines, giving rising rates of NCDs across Asia
Pharmacists	• Continue to encourage through education and other approaches/recommended strategies to prevent the spread of COVID-19 including preventative measures such as PPE. This includes providing education about COVID-19 and ways to reduce the spread of the virus which includes corporate responsibilities • Through stock control and other measures, try and ensure PPE and pertinent medicines, including suitable alternatives, that have proven to be beneficial for patients with COVID-19 are routinely available, and help ensure where possible that any price rises are kept to a minimum especially in countries with high co-payment levels • Encourage patients to seek testing and medical advice where COVID-19 is suspected, building on current strategies in Malaysia and Vietnam (Table 2), e.g., in Vietnam pharmacists are requested to ask patients buying medicines for acute respiratory infections such as coughs, fever, shortness of breath and colds to make a health declaration (53) • This is important since it can be difficult in practice to differentiate respiratory tract infections from COVID-19 in patients presenting with coughs and fever (225). As part of this, where pertinent, continue to argue/council patients against the need for antibiotics where concerns • Work with patients to enhance adherence to medicines especially those for NCDs where it can be difficult for patients to attend clinics • Potentially become involved in vaccination programmes where there is unmet need (226)
Patients/Patient organizations	• Where possible, engage with social media and other key channels to promote evidence-based approaches to the prevention and management of COVID-19 given the current extent of misinformation to date, and ensure messages are as clear as possible and in a positive language (224) • In conjunction with this, seek to work with governments and other key stakeholder groups to minimize the impact of any misinformation (224) alongside working with them to seek to reduce the consequences of COVID-19 on mental health issues including any associated stigma • Continue educating patients through various channels. This includes the importance of self-management and adherence to prescribed treatments in patients with chronic NCDs – given the challenges of clinic attendance arising from lockdown measures. Explore new technologies including telemedicine and other approaches to reduce reliance on clinic attendance especially during pandemics as well as among rural patients in LMICs

*CVD, cardiovascular disease; LMICs, low- and middle-income countries; NB: NCDs, non-communicable diseases; PPE, personal protective equipment*.

## Discussion

We believe this is one of the first studies to assess the impact of COVID-19 on the utilization, availability and price changes of pertinent medicines and PPE to prevent and treat patients with COVID-19 among Asian countries in the early stages of the pandemic. This is important as payments for medicines among a number of these LMICs can potentially be catastrophic for patients, and spending valuable resources on unproven medicines diverts scarce monies away from funding medicines in priority disease areas. Considerable increases in the prices of vitamins/immune boosters and PPE is also a concern where this exists (Table 5), again diverting monies away from funding treatments in other priority disease areas, probably reflecting some of the shortages seen (Table 6). Greater monitoring of prices as well as increases in local production of medicines and PPE may be ways forward to reduce future price rises and shortages (62), and we will continue to monitor this building on ongoing initiatives among African and Asian countries (Table 1) (61).

It was encouraging to see appreciable increases in the utilization of PPE as well as Vitamin C/immune boosters across the studied countries (Table 4) suggesting that public health messages are getting through, building on positive experiences in Pakistan (10, 90). Alongside this, it was encouraging to see no change or decreased utilization of antimalarials and antibiotics in an appreciable number of pharmacies in India, Malaysia and Pakistan, similarly for antimalarials in Bangladesh. This is in line with initiatives by governments certainly in India, Malaysia, and Vietnam to try and restrict the sales of antimicrobials to reduce resistance development, which we see continuing. There are a number of initiatives that can be undertaken in Bangladesh and Pakistan to enhance the appropriate use of antimicrobials. This includes providing increased education and guidelines to pharmacists where there are concerns (129, 227), as well as greater patient education. In addition, greater enforcement of any legislation banning the dispensing of antimicrobials without a prescription. Such activities have worked well in other countries including other LMICs (125, 132, 228–230) without the need for fines, which can be problematic and difficult to enforce (223). This is important in patients with COVID-19 since, as mentioned (Table 7), it can be difficult in practice to differentiate respiratory tract infections from COVID-19 in patients presenting with coughs and fever (225). Consequently, early referral for testing and subsequent management is encouraged where possible.

The increased use of analgesics across a number of the countries studied is also in line with expectations (Table 4), and as mentioned, we would expect increased utilization of vitamins/immune boosters and PPE to continue if COVID-19 infection rates increase following any easing of lockdown measures.

Table 7 highlights potential activities that can be undertaken by key stakeholder groups going forward including addressing unintended consequences. A key area is the level of misinformation seen especially surrounding chloroquine and hydroxychloroquine with potentially devastating consequences (193, 196, 231, 232). Consequently, as mentioned, there is a need to ensure that recommended strategies from Minsteries of Health and leading physician and pharmacy organizations are evidence based given the extent of redactions and concerns that have been seen to date (161, 174, 191, 192). Another key area from a public health viewpoint is addressing the unintended consequences of lockdown measures. This includes an increase in other infectious diseases if immunization programmes and other measures are not undertaken as well as an increase in mental health disorders and other NCDs as a result of the pandemic (99, 100, 198, 200, 201). We are aware that lockdown measures in sub-Saharan Africa could result in up to 18 million additional cases of malaria and up to 30,000 additional deaths (233–235), with similar concerns in infected Asian countries. There are also considerable concerns with appreciably increased mortality in other infectious diseases if children are not being vaccinated (236). Consequently, avoided if possible. Telemedicine and other approaches can help with mental health concerns as well as concerns with other NCDs in LMICs (199), and this is likely to continue. However, mindful that any approach needs to take into account individual patient's needs and their specific situation (237).

Community pharmacists are likely to have an increasing role in the future across countries including LMICs as their knowledge and experience grows. This includes potentially instigating educational and other programmes to enhance adherence to medicines in patients with chronic NCDs given ongoing concerns as well as give guidance and public health education on prevention and possible treatments for patients with suspected COVID-19 (4, 128, 202–205). Their potential role also includes helping with immunization programmes with studies showing that when pharmacists provide immunizations, they substantially increase rates (226). Community pharmacists can also push for extended prescription lengths where this is a concern and patients have difficulties with obtaining supplies. We will be exploring the unintended consequences in future research projects as these considerations are important in future planning.

## Limitations

We are aware of a number of limitations with this study. These include the fact that we were only able to undertake pilot studies in a number of countries including Pakistan and Vietnam. In addition, we were unable to obtain exact details on changes in the utilization and prices of pertinent medicines and PPEs from all the pharmacists visited due to issues of confidentiality and having the data readily to hand. We were also unable to undertake any time series analysis as we were primarily interested in changes post the pandemic, and this was not broken down by month. As a result, we did not adjust for any seasonality. However, we are confident our findings and ways forward for all key stakeholder groups can be helpful for future planning purposes including extending the role of community pharmacists across countries.

## Conclusion

We have seen increases in utilization and prices for antimalarials and antibiotics across countries arising from the COVID-19 pandemic, with considerable increases in some countries. This needs addressing through educational and other activities to prevent rises in resistance rates. Community pharmacies and others, including patient organizations, can also play a key role with improving prevention measures as well as reducing the impact of any misinformation given the consequences experienced among countries.

Key stakeholder groups including community pharmacists and patient organizations can also help address the unintended consequences from lockdown and other activities including potential increases in infectious diseases and greater morbidity from NCDs, and we will be monitoring this in the future. Encouragingly, there was increased use of vitamins/immune boosters and PPE among the Asian countries. However, the considerable price rises seen are a concern in countries with existing high co-payment levels and no government control on prices, which need addressing. Community pharmacists can again play a role here alongside the Government.

## Data Availability Statement

The raw data supporting the conclusions of this article will be made available by the authors, without undue reservation.

## Ethics Statement

Ethical approval for this study was not required according to national legislation and institutional guidelines. However, as before, all pharmacists freely provided the requested information having been given the opportunity to refuse to participate if wished.

## Author Contributions

All authors listed have made a substantial, direct and intellectual contribution to the work, and approved it for publication.

## Conflict of Interest

AR, MK, and MonH were employed by Grameen Euglena, Al-Manar Hospital Ltd. & Modern Hospital Cumilla Ltd., and Square Toiletries Limited, Dhaka, Bangladesh, respectively. The remaining authors declare that the research was conducted in the absence of any commercial or financial relationships that could be construed as a potential conflict of interest.
